# Is the Anterolateral or Posterolateral Approach More Effective for Early Postoperative Recovery after Minimally Invasive Total Hip Arthroplasty?

**DOI:** 10.3390/jcm12010139

**Published:** 2022-12-24

**Authors:** Yoichi Ohta, Ryo Sugama, Yukihide Minoda, Shigekazu Mizokawa, Shinji Takahashi, Mitsuhiko Ikebuchi, Tamotsu Nakatsuchi, Hiroaki Nakamura

**Affiliations:** 1 Department of Orthopaedic Surgery, Osaka Metropolitan University Graduate School of Medicine, Osaka 545–8585, Japan; 2 Tsuji-geka Rehabilitation Hospital, Osaka 543–0072, Japan

**Keywords:** minimally invasive total hip arthroplasty, postoperative recovery, surgery

## Abstract

Currently, various minimally invasive surgical techniques are applied for total hip arthroplasty (THA). There are few studies comparing the early postoperative clinical outcomes of minimally invasive THA between anterolateral and posterolateral approaches. In this retrospective study, 62 patients underwent minimally invasive THA via either the anterolateral approach with an intermuscular exposure using the modified Watson–Jones approach (MIS-AL, 34 hips) or mini-incision THA with a posterolateral approach (MIS-PL, 28 hips). We analyzed intraoperative data, postoperative hematological data, postoperative radiographic findings, and the postoperative recovery of muscle strength. The mean surgical time was significantly longer in the MIS-PL than in the MIS-AL group. The mean postoperative serum C-reactive protein level was significantly higher in the MIS-PL group than in the MIS-AL group only on postoperative day 3. There were no significant between-group differences in the postoperative recovery rate of muscle strength during hip abduction. The recovery rate of muscle strength during hip extension was better in the MIS-AL group than in the MIS-PL group only on postoperative day 3. In conclusion, we found no obvious advantage in early postoperative recovery between the MIS-AL and MIS-PL approaches. Therefore, the benefit of rapid postoperative recovery was comparable between the MIS-AL and MIS-PL approaches.

## 1. Introduction

Minimally invasive surgical approaches have been developed in many fields of surgery; these approaches are associated with a greater need for early return to work in some patient populations, shorter hospital stays, and a reduced cost of management [1,2,3].

Minimally invasive total hip arthroplasty (THA) has the following benefits: reduced soft tissue damage around the hip joint, shorter intraoperative time, less perioperative blood loss, and more rapid rehabilitation of patients after surgery [4,5,6]. Nevertheless, one of the disadvantages of minimally invasive approaches is low intraoperative visibility, which has a risk of component malpositioning, thereby affecting long-term clinical outcomes [7,8,9,10,11].

Currently, various minimally invasive surgical techniques are applied for THA and hemiarthroplasty [4,12,13,14,15,16]. There are few studies comparing the early postoperative clinical outcomes of minimally invasive THA between anterolateral and posterolateral approaches. The minimally invasive anterolateral technique using the modified Watson–Jones approach for THA reportedly spares the abductors, external rotators, and posterior capsule. Therefore, it guarantees rapid postoperative rehabilitation management through the preservation of muscle integrity [4]. Inaba et al. reported that the muscle strength recovery of hip abduction 6 weeks after surgery using the modified Watson–Jones approach was better than the mini-incision direct lateral approach [9]. Furthermore, they demonstrated that the serum creatinine kinase level at postoperative day 1 was lower in patients who underwent the modified Watson–Jones approach than in those who underwent the mini-incision direct lateral approach [9]. Martin et al. showed that patients who underwent THA using the modified Watson–Jones approach had less operative blood loss [17]. In addition, some reports described a shorter surgical time, reduced perioperative blood loss, and earlier recovery with the posterior mini-incision approach for THA [13,18,19].

Wenz et al. reported that the mini-incision THA with the posterior approach had less mean surgical time and blood loss. Moreover, they demonstrated that patients with posterior mini-incision THA had less intraoperative blood transfusion requirements and earlier postoperative functional recovery [18]. The posterior approaches for MIS-THA require posterior capsulotomy; thus, they traditionally increase postoperative dislocation risk [10]. Weeden et al. reported that the posterior approach for THA can result in an extremely low dislocation rate with enhanced soft tissue repair [20].

We hypothesized that the early postoperative recovery of hip muscle strength could be achieved more consistently by using the muscle-sparing anterolateral approach than the mini-incision posterolateral approach. This study aimed to compare two types of minimally invasive surgical approaches for THA: the anterolateral and posterolateral approaches; moreover, we assessed whether each approach had a beneficial effect on early postoperative recovery after THA.

## 2. Materials and Methods

We investigated 62 patients with no prior surgery on the affected hips, who underwent minimally invasive THA. The inclusion criteria were patients who were able to measure muscle strength during hip extension and abduction before and after THA. The patients were classified into two groups based on the surgical approach used: the anterolateral approach with a muscle-sparing technique using the intermuscular plane between the gluteus medius and the tensor fascia lata (MIS-AL group, 34 hips) and mini-incision THA with the posterolateral approach (MIS-PL group, 28 hips). Operations were performed by two senior joint surgeons in the same joint surgical team. The approach was decided based on the surgeon’s preference.

In the MIS-AL group, the operation was performed using a modified Watson–Jones approach developed by Rottinger et al. [4]. By comparison, in the MIS-PL group, the surgery was performed through an 8–10 cm incision using a posterolateral minimally invasive approach. The gluteus maximum was split, and the short external rotators and capsule were taken as a unit. The short external rotators and capsule were repaired to the greater trochanter after implantation [21].

All the patients in both groups underwent THA with an identical clinical pathway in our hospital. They were aimed for discharge at postoperative week 3. Physical therapy started, and the patients were allowed to bear full weight using a walker on the first postoperative day.

The recorded preoperative clinical data included age, sex, diagnosis, and body mass index. Intraoperative data included the surgical time. Intraoperative blood loss was estimated. Serum hemoglobin, hematocrit, creatine kinase, and C-reactive protein levels were assessed for each patient on postoperative day 1, day 3, week 1, and week 2. Baseline hemoglobin and hematocrit levels were measured preoperatively.

We measured the cup inclination and anteversion angles on the anteroposterior radiograph using the methods of Lewinnek et al. [22]. Stem alignment was assessed if the angle between the long axis of the stem and the anatomical axis of the femur on the anteroposterior radiograph was more than 3° in varus or valgus alignment [21,23]. All the radiographic measurements were performed by an experienced orthopedic surgeon who was not one of the operating surgeons in this study using SYNAPSE (FUJIFILM Medical System, Stamford, CT, USA).

We evaluated the number of postoperative days patients could walk >150 m with a single assistive walking device.

We measured the postoperative recovery rate of muscle strength during hip extension and abduction before surgery and at postoperative day 3, day 7, day 10, and day 14 using a hand-held dynamometer (J-Tech Medical, Power Track II). The ratio of muscle strength before surgery to that after surgery at each follow-up visit was calculated.

This study was approved by the institutional review board of Osaka City University (approval number 1280). The study had a retrospective design, and thus an opt-out method was used instead of informed consent.

The sample size was calculated based on our data on hip extension strength on day 3 after THA. The recovery rates of MIS-AL and MIS-PL were estimated as 60 ± 40 and 40 ± 20, respectively. The overall sample size of 54 with 90% power at a 0.05 significance level to detect the difference using a *T*-test.

A *T*-test was used for continuous variables. The Shapiro–Wilk test was used to assess the normality of the data. Fisher’s exact test was used for categorical variables. A mixed effects model was used to analyze the repeated measures of postoperative hematological data and muscle strength across the 2 weeks following surgery. Regarding serum hemoglobin and hematocrit, the Dunnett test was used to compare the preoperative and postoperative values. A *p*-value < 0.05 was statistically significant. All the *p*-values were two-sided. All analyses were performed using SAS version 9.4 (SAS Institute, Inc., Cary, NC, USA).

## 3. Results

Table 1 shows the preoperative demographic data and preoperative diagnosis. There were 34 women in the MIS-AL group and 28 women in the MIS-PL group. The mean participant ages were 62.8 ± 9.6 and 65.3 ± 10.9 years in the MIS-AL and MIS-PL groups, respectively. The mean body mass indexes were 23.2 ± 3.1 and 24.0 ± 3.4 kg/m^2^ in the MIS-AL and MIS-PL groups, respectively. There were no significant between-group differences in demographic parameters. The mean surgical time in the MIS-PL group (99.4 ± 20.0 min) was significantly longer than that in the MIS-AL group (82.7 ± 18.3 min) (*p* < 0.05) (Table 2). The mean perioperative blood loss was 249.7 ± 200.5 mL in the MIS-AL group and 228.0 ± 141.9 mL in the MIS-PL group (*p* = 0.63) (Table 2).

The mean postoperative serum hemoglobin and hematocrit levels were significantly lower than the preoperative baseline in the MIS-AL and MIS-PL groups (*p* < 0.05).

The mean postoperative serum hemoglobin and hematocrit levels on day 1, day 3, week 1, and week 2 were not significantly different between the MIS-AL and MIS-PL groups (Table 2). Further, there were no significant between-group differences in the mean postoperative serum levels of creatine kinase. C-reactive protein level on day 3 was significantly higher in the MIS-PL than in the MIS-AL group, whereas there were no significant between-group differences in the C-reactive protein levels on day 1, week 1, and week 2 (Table 2).

The mean cup inclination angle was 43.2 ± 7.6° in the MIS-AL group and 42.0 ± 4.9° in the MIS-PL group (*p* = 0.46). The mean cup anteversion angle was 17.5 ± 6.1° in the MIS-AL group and 14.3 ± 7.0° in the MIS-PL group (*p* = 0.06). (Table 3). In the MIS-AL group, 2 and 32 hips were in varus and neutral alignments, respectively, whereas all 28 were in neutral alignment in the MIS-PL group (Table 3).

The patients could walk more than 150 m with a single assistive walking device on postoperative days 8.5 ± 5.1 and 9.7 ± 3.5 in the MIS-AL and MIS-PL groups, respectively (*p* = 0.32) (Table 4).

There were no between-group differences in the postoperative recovery rate of muscle strength during hip abduction on day 3, day 7, day 10, and day 14 (Table 5). The postoperative recovery rate of muscle strength during hip extension was significantly better in the MIS-AL group only on postoperative day 3; however, there were no significant between-group differences on day 7, day 10, and day 14 (Table 5).

## 4. Discussion

Minimally invasive techniques in THA have received widespread attention in recent years because of their considerable benefits. In developing less invasive approaches for THA, both anterior and posterior approaches have been used. In this study, we evaluated the postoperative recovery of two minimally invasive techniques (MIS-AL and MIS-PL approaches) in the postoperative period.

The MIS-AL preserves the posterior capsule and short rotators [4]; however, there are concerns about the increased risk of postoperative dislocation after the posterior approaches for MIS-THA [10]. To combat this problem, we performed a repair of the capsule and short external rotators after inserting the implants; there was no dislocation in patients of both the MIS-AL and MIS-PL groups. In this study, the surgical time was longer in the MIS-PL than in the MIS-AL group, probably due to the additional procedure of repairing the posterior structures during the MIS-PL approach.

Superior gluteal nerve damage may occur during the anterior approach [24,25,26], although inferior gluteal nerve damage may also occur during the posterior THA approach [27] with a similar frequency. The postoperative muscle strength during hip abduction and hip extension increased from the early postoperative days in both the MIS-AL and MIS-PL groups in this study. As a result of the advantage of preserving the abductor’s muscle, it was considered that the postoperative muscle strength of the abductor’s muscles recovered early after surgery in the MIS-AL group as in the MIS-PL group. The postoperative recovery rate of the extensor muscle was lower in the MIS-PL group than in the MIS-AL group only on postoperative day 3. In addition, the mean postoperative serum level of C-reactive protein on day 3 was significantly higher in the MIS-PL group than in the MIS-AL group. C-reactive protein has been used to evaluate the soft tissue impairment of different approaches in THA [28,29]. These differences seemed to be due to the splitting of the gluteus maximum during the MIS-PL approach. The mean incision length measured 7.91 ± 0.63 cm and 8.34 ± 0.64 cm in the MIS-AL and MIS-PL groups (*p* < 0.05), respectively. The MIS-PL incision length was significantly longer than that of the MIS-AL. As the postoperative rise in serum C-reactive protein indicates inflammation level, the higher mean postoperative serum level of C-reactive protein elevation on day 3 in MIS-PL might also be related to the longer surgery time and incision length. As there were no differences in the postoperative recovery rate of muscle strength during hip extension between both approaches after postoperative day 7, we considered the abovementioned difference not clinically significant. There were no between-group differences in the postoperative recovery rate of muscle strength during both hip abduction and extension at month 3 (hip abduction, 129.7 ± 34.5% and 151.5 ± 55.3%, *p* > 0.05; hip extension, 137.1 ± 70.3% and 134.6 ± 69.4%, *p* > 0.05 in the MIS-AL and MIS-PL groups, respectively) and month 6 (hip abduction, 137.9 ± 46.7% and 164.2 ± 53.6%, *p* > 0.05; hip extension, 141.8 ± 77.6% and 147.9 ± 74.7%, *p* > 0.05 in the MIS-AL and MIS-PL groups, respectively). Considering the results of the mean postoperative serum levels of creatine kinase and C-reactive protein for soft tissue damage after postoperative week 1, there were no apparent symptoms of damage to the abductor and extensor muscles in both the MIS-AL and MIS-PL groups. Lin et al. reported that patients with mini-incision anterolateral THA had significantly better hip muscle strength and walking speed [30]. Inaba et al. reported that the muscle strength recovery of hip abduction was better at 6 weeks after surgery in the group using the muscle-sparing MIS approach (modified Watson–Jones approach) than in the group using the modified mini-incision direct lateral approach [9]. In this study, although we compared the mean postoperative period during which patients were capable of walking >150 m with a single assistive walking device, there was no superiority in the MIS-AL group.

Component positioning was almost within the desired range in both groups. No differences in the inclination angle and anteversion angle of the acetabular component were observed between the MIS-AL and MIS-PL groups. Regarding stem alignment, Teet et al. reported that a significantly increased proportion of stems were aligned in the varus direction from the neutral position through the MIS-PL approach compared with the classical posterolateral approach [31]. In this study, there was no varus or valgus alignment in the MIS-PL group; however, two patients demonstrated varus alignment in the MIS-AL group. This may be due to the technical difficulties related to femoral canal visualization during the MIS-AL approach.

There were several limitations in this study. First, this study was not a randomized clinical trial. Second, this study was retrospective in nature. Third, the approach was decided based on the surgeon’s preference. Fourth, the hip prostheses used in this study were not identical; the inclusion criteria were patients for whom muscle strength could be measured before and after THA. Due to these limitations, it is difficult to completely exclude the possibility of selection and surgeon bias in this study.

We hypothesized that the early postoperative recovery of hip muscle strength could be superior by using the muscle-sparing anterolateral approach than the mini-incision posterolateral approach. The recovery rate of muscle strength during hip extension was better in the MIS-AL than in the MIS-PL group only on postoperative day 3. In this study, although we could not demonstrate the relevant superiority in the early postoperative recovery period between the MIS-AL and MIS-PL approaches, the use of these two approaches preserved muscle integrity; moreover, they were useful for the early recovery of postoperative function in patients who underwent THA.

## Figures and Tables

**Table 1:** Patient demographic data according to MIS-AL or MIS-PL minimally invasive THA approach.

	MIS-AL	MIS-PL	*p*-Value
No. of hips	34	28	
Age (y) *	62.8 ± 9.6	65.3 ± 10.9	0.35
Sex (M/F)	0/34	0/28	1
Body mass index *	23.2 ± 3.1	24.0 ± 3.4	0.31
Diagnosis (no. of patients)			
Osteoarthritis	34	28	

* Values are expressed as the mean and standard deviation. Abbreviation: MIS-AL, anterolateral approach with a muscle-sparing technique using the intermuscular plane between the gluteus medius and the tensor fascia lata; MIS-PL, mini-incision total hip arthroplasty with the posterolateral approach.

**Table 2:** Intraoperative data and postoperative hematological data.

	MIS-AL	MIS-PL	*p*-Value
Surgical time (min) *	82.7 ± 18.3	99.4 ± 20.0	<0.05
Intraoperative blood loss (mL) *	249.7 ± 200.5	228.0 ± 141.9	0.63
Hemoglobin (nmol/L)			
Preop	11.8 ± 1.0	12.2 ± 1.3	0.21
at 1 day *^,†^	11.1 ± 0.9	11.1 ± 1.3	0.61
at 3 days *^,†^	11.2 ± 1.0	11.3 ± 1.3	0.92
at 1 week *^,†^	11.0 ± 1.1	10.9 ± 1.2	0.67
at 2 weeks *^,†^	11.3 ± 0.9	11.3 ± 1.2	0.96
Hematocrit			
Preop	36.5 ± 2.8	37.2 ± 3.4	0.38
at 1 day *^,†^	33.9 ± 2.8	34.0 ± 3.9	0.67
at 3 days *^,†^	34.9 ± 3.5	34.6 ± 4.0	0.80
at 1 week *^,†^	34.1 ± 3.6	33.5 ± 3.5	0.59
at 2 weeks *^,†^	35.3 ± 2.7	35.1 ± 3.3	0.85
Creatine kinase (IU/L)			
at 1 day *	652.4 ± 236.6	641.8 ± 516.6	0.14
at 3 days *	427.6 ± 316.7	570.9 ± 485.9	0.26
at 1 week *	168.4 ± 108.9	187.9 ± 151.0	0.89
at 2 weeks *	54.7 ± 23.1	57.6 ± 18.1	0.94
C-reactive protein (mg/dL)			
at 1 day *	4.2 ± 1.8	4.8 ± 2.2	0.32
at 3 days *	11.0 ± 5.6	12.5 ± 4.6	<0.05
at 1 week *	3.0 ± 1.8	3.5 ± 1.9	0.41
at 2 weeks *	0.8 ± 0.9	0.9 ± 0.8	0.96

* Values are expressed as the mean and standard deviation; ^†^ Postoperative values are significantly different from baseline.

**Table 3:** Postoperative radiographic findings.

	MIS-AL	MIS-PL	*p*-Value
Cup inclination (°) *	43.2 ± 7.6	42.0 ± 4.9	0.46
Cup anteversion (°) *	17.5 ± 6.1	14.3 ± 7.0	0.06
Stem alignment on Anteroposterior radiograph			
Neutral	32	28	0.50
Varus (>3°)	2	0	
Valgus (>3°)	0	0	
Total	34	28	

* Values are expressed as the mean and standard deviation.

**Table 4:** Postoperative days wherein patients were capable of walking for more than 150 m with a single assistance walking device.

	MIS-AL	MIS-PL	*p*-Value
Postoperative days *	8.5 ± 5.1	9.7 ± 3.5	0.32

* Values are expressed as the mean and standard deviation.

**Table 5:** Comparison of pre- versus postoperative recovery rates of muscle strength.

	MIS-AL	MIS-PL	*p*-Value
Hip abduction			
at 3 days (%) *	59.5 ± 27.8	63.1 ± 34.8	0.69
at 7 days (%) *	78.8 ± 31.2	89.6 ± 44.7	0.15
at 10 days (%) *	89.1 ± 31.9	99.6 ± 42.8	0.23
at 14 days (%) *	102.7 ± 39.2	115.8 ± 50.9	0.16
Hip extension			
at 3 days (%) *	59.2 ± 39.2	39.5 ± 20.8	<0.05
at 7 days (%) *	82.6 ± 49.4	62.4 ± 45.5	0.06
at 10 days (%) *	86.8 ± 41.3	73.1 ± 34.7	0.19
at 14 days (%) *	98.2 ± 43.1	82.1 ± 38.6	0.12

* Values are expressed as the mean and standard deviation.

## Data Availability

The datasets generated and/or analyzed during the current study are available from the corresponding author upon reasonable request.
